# Evaluation of clinical and sociodemographic characteristics of hospitalised patients with schizophrenia spectrum disorder

**DOI:** 10.1192/j.eurpsy.2024.1519

**Published:** 2024-08-27

**Authors:** H. R. Demirel, E. Yıldız, S. N. İspir, M. Aydın

**Affiliations:** Psychiatry, Selçuk University Faculty Of Medicine, Konya, Türkiye

## Abstract

**Introduction:**

Schizophrenia and other psychotic disorders are disorders in which the individual’s assessment of reality is impaired and which progress with exacerbations and become chronic, leading to disability, loss of function, social communication problems and frequent hospitalisations.

**Objectives:**

The aim of our study was to evaluate the clinical and sociodemographic data of patients followed up in the outpatient clinic for psychotic disorders and hospitalized at least once in any time during their treatment.

**Methods:**

The sample of the present study consisted of patients who were followed up in the psychotic disorders outpatient clinic of Selçuk University Faculty of Medicine Hospital and who were hospitalised at least once. Patients were identified by retrospective file search and those with sufficient information about their sociodemographic-clinical characteristics were included. The study approved by the ethics committee of Selçuk University Faculty of Medicine.

**Results:**

Of the 130 patients, 52 (40%) were female and 78 (60%) were male; mean age was 40.8 ± 12.0 years. Almost half of the patients (n=53, 40.8%) had primary school education. 73 (59.2%) of 130 patients were receiving long-acting antipsychotic medication. 100 patients (76.9%) were using oral antipsychotics. 63 out of 100 patients were on clozapine. 22 of 63 patients used clozapine as monotherapy. The mean duration of untreated psychosis (n=90) was 15.8 ± 32.1 months. The mean number of hospitalisations was 3.4 ± 2.5. 15 patients (11.5%) were lived in a nursing home. The mean number of hospitalisations of patients receiving long-acting treatment (3.8±2.9) was significantly higher than that of patients receiving oral treatment only (2.7±1.6) (p=0.004). There was no significant difference in the mean number of hospitalisations when comparing according to the presence of clozapine in the treatment (p>0.05).

**Conclusions:**

The primary goal in the treatment of patients with schizophrenia is to prevent relapses, hospital admissions and improve patients’ quality of life and functioning. Therefore, the variables related to hospitalisations, which are an indirect indicator of the frequency of psychotic episodes, should be well evaluated. Our study was mainly descriptive and evaluated the relationship between several parameters and hospitalisations. It was thought that the high number of hospitalisations in patients on long-acting treatment might be related to the fact that long-acting treatment in our country is mostly started in the late stages of the disease. Large-sample studies of predictive parameters are needed to prevent psychotic episodes and reduce the number of hospitalisations.

**Disclosure of Interest:**

None Declared

